# Intra-annual stem radius growth and cell formation of two diffuse-porous tree species in a subtropical forest in Southwest China

**DOI:** 10.1093/treephys/tpaf020

**Published:** 2025-02-17

**Authors:** Yi-Xue Zhang, Pei-Li Fu, Qiao-Shun Yan, Achim Bräuning, Ze-Xin Fan

**Affiliations:** CAS Key Laboratory of Tropical Forest Ecology, Xishuangbanna Tropical Botanical Garden, Chinese Academy of Sciences, Menglun, Mengla, Yunnan 666303, China; College of Life Sciences, University of Chinese Academy of Sciences, Yuquan Road 19A, Beijing 100049, China; CAS Key Laboratory of Tropical Forest Ecology, Xishuangbanna Tropical Botanical Garden, Chinese Academy of Sciences, Menglun, Mengla, Yunnan 666303, China; CAS Key Laboratory of Tropical Forest Ecology, Xishuangbanna Tropical Botanical Garden, Chinese Academy of Sciences, Menglun, Mengla, Yunnan 666303, China; Ailaoshan Station of Subtropical Forest Ecosystem Studies, Xishuangbanna Tropical Botanical Garden, Chinese Academy of Sciences, Taizhong, Jingdong, Yunnan 676209, China; Institute of Geography, Friedrich-Alexander-University Erlangen-Nürnberg, Wetterkreuz 15, Erlangen 91058, Germany; CAS Key Laboratory of Tropical Forest Ecology, Xishuangbanna Tropical Botanical Garden, Chinese Academy of Sciences, Menglun, Mengla, Yunnan 666303, China; Ailaoshan Station of Subtropical Forest Ecosystem Studies, Xishuangbanna Tropical Botanical Garden, Chinese Academy of Sciences, Taizhong, Jingdong, Yunnan 676209, China

**Keywords:** dendrometer, intra-annual growth, subtropical evergreen forest, seasonality, vessels, xylogenesis

## Abstract

Studying tree growth and xylem formation is essential for understanding tree resilience to extreme droughts, which are expected to intensify with climate warming. However, researches on intra-annual stem growth and xylogenesis remain limited, particularly in moist subtropical forests. This study monitored the intra-annual stem radius growth and xylem formation of two diffuse-porous tree species, *Stewartia pteropetiolata* W. C. Cheng and *Schima noronhae* Reinw. ex Blume, in a subtropical evergreen broadleaved forest in Southwest China, using high-resolution dendrometer measurements for recording stem growth and micro-coring for xylem formation. We analyzed the seasonal patterns of stem radius growth and xylem formation and their responses to seasonal climate variability. Our results revealed that *S. noronhae*, found at lower elevations, exhibited a later onset of stem growth and xylogenesis, developing wider vessels with thinner walls during a longer enlarging phase. In contrast, *S. pteropetiolata*, which is distributed at higher elevations, produced smaller vessels with thicker walls during a longer cell-wall thickening phase. Both species showed high relative growth rates under conditions of high temperatures and low vapor pressure deficit. More specifically, *S. noronhae* maintained higher relative growth rates under a narrower range of favorable temperature and soil water conditions during the rainy season, while *S. pteropetiolata* sustained growth for a longer growth period in colder and drier conditions. These findings enhance the understanding of angiosperm wood cell kinetics and the eco-physiological response of diffuse-porous trees to climate change in moist subtropical forests.

## Introduction

The frequency and intensity of extreme climate events, such as droughts and heat waves, have increased significantly over the past few decades (Coumou and Rahmstorf 2012, AghaKouchak et al. 2020) and are projected to continue rising due to climate change (Fischer and Knutti 2014, Lee et al. 2023). Tree growth is profoundly influenced by climatic variability such as extreme temperatures and atmospheric vapor pressure deficit (VPD) (Way and Oren 2010, Zweifel et al. 2021). Information on intra-annual growth and xylem formation of tree species across diverse climatic gradients, as well as their responses to environmental variability, is crucial for understanding the responses of forest ecosystems to climate change (Steppe et al. 2015, Martínez-Sancho et al. 2022). Furthermore, understanding cambial phenology and quantitative wood anatomy is essential for elucidating how xylogenesis adjusts to climate changes (Pérez-de-Lis et al. 2016, Prislan et al. 2018, Zhang et al. 2019). Despite their importance, relevant studies in moist subtropical forests remain limited.

Cambial activity, the process by which the cambium produces new xylem and phloem tissues, is directly influenced by short-term environmental conditions (Etzold et al. 2022). While cell production may accelerate with rising spring temperatures (Körner and Paulsen 2004, Rossi et al. 2007), excessive heat can inhibit metabolic activity and reduce cambial activity in the later development stages (Parent et al. 2010, Ruehr et al. 2016). Following the cambial activity, xylogenesis results in the maturation of functional xylem cells and determines the wood quality and plant physiology through anatomical features (Hughes et al. 2010). Wood anatomical features in angiosperms involve a tradeoff between hydraulic efficiency and safety, which is crucial for maintaining water balance (Wheeler et al. 2005, Brodribb 2009). In humid temperate forests, particularly at higher elevations, freeze–thaw cycles threaten the hydraulic function in overwintering organs of woody species (Choat et al. 2011, Christensen-Dalsgaard and Tyree 2013, Granda et al. 2014, Araujo et al. 2023). Severe droughts can induce xylem embolisms, impairing water transport and reducing tree growth (Hoffmann et al. 2011, Pivovaroff et al. 2016). Integrating cambial phenology with wood anatomical traits is essential for gaining deeper insights into tree adaptation to climate change (Li et al. 2021).

Stem radial growth dynamics mainly consists of two components: stem growth phenology including the initiation, termination and duration of the growth period and stem growth activity including the growth rate and the number of days with active growth (Etzold et al. 2022). Both cambial activity and xylogenesis are strongly influenced by atmospheric VPD and soil water availability, ultimately affecting the radial growth dynamics of trees (Körner 2003, Cabon et al. 2020*a*, Zweifel et al. 2021). Most studies on the cambium activity of angiosperm tree species focused on xylem width at different developmental stages or the number of cambial cells (Prislan et al. 2013, Pérez-de-Lis et al. 2017). However, our understanding of the timings and kinetics of xylem formation in different cell types (e.g. fiber cells, vessels) of angiosperm trees remains limited. Studying the radial growth dynamics and formation of different cell types in diffuse-porous tree species will enhance our understanding of how trees ecophysiologically may respond to future climate change.

Our study investigated intra-annual stem growth and xylem formation in two diffuse-porous tree species, *Stewartia pteropetiolata* W. C. Cheng and *Schima noronhae* Reinw. ex Blume. These two species are dominant and coexist in the subtropical evergreen forest in the Ailao Mountain in Southwest China. We addressed two questions. (i) What are the differences in intra-annual patterns of stem growth and xylogenesis between the two species? (ii) What climatic factors most strongly influence stem growth and xylogenesis, particularly vessel and fiber development?

## Materials and methods

### Study site and species

The study site is located in a subtropical evergreen broad-leaved forest in the Ailao Mountains (24°32′N, 101°01′E, 2460 m a.s.l.), Yunnan Province, Southwest China. The regional climate is strongly influenced by the Indian summer monsoon, which typically lasts from June to September. The mean annual temperature is 11.7 °C, with the lowest mean monthly temperature occurring in January (5.3 °C) and the highest in July (15.3 °C). Annual precipitation is 1728 mm, with 86% of the rainfall occurring during the rainy season from May to October, leading to a distinct dry season from November to April (Wu et al. 2014, Fei et al. 2018).

Meteorological data were recorded every 10 min in 2020 at the Ailao Station for Subtropical Forest Ecosystem Studies, which is located 500 m away from our study site. The raw climate data were aggregated into daily averages, including minimum air temperature (°C), maximum air temperature (°C), mean air temperature (°C), relative humidity (RH, %), mean photosynthetically active radiation (mol m^−2^ s^−1^) and volumetric soil water content (SWC, m^3^ m^−3^). Considering that roots are most abundant near the soil surface (Nepstad et al. 1994, Jackson et al. 1996), SWC measured at 10 cm depth was used for further analyses (Etzold et al. 2022, Martínez-Sancho et al. 2022). The VPD (kPa) was calculated based on air temperature and RH by R package ‘*plantecphys*’ (Duursma 2015).

The forest in the study area is primarily dominated by evergreen broadleaved tree species from the Fagaceae, Theaceae, Magnoliaceae and Lauraceae families. Evergreen and deciduous broadleaved tree species account for 76.2 and 22.8% of the 101 woody species recorded in a 20-ha permanent plot (Han-Dong et al. 2018). Theaceae species are the most abundant, representing 23.7% of the total individuals in the plot. Within the Theaceae family, *Stewartia pteropetiolata* (SP) and *Schima noronhae* (SN) are the dominant evergreen canopy species (Wen et al. 2018). For each species, five individuals were selected for micro-coring, with three of them equipped with high-resolution dendrometers.

### Stem radius variations

Stem radius variations (SRV) were monitored using point dendrometers (DR type, Ecomatic, Germany) with a spatial resolution of 2 μm. Dendrometers were installed at breast height on three trees of each species. The mean diameters of *S. pteropetiolata* and *S. noronhae* were 29.6 and 33.6 cm, respectively. To minimize the potential effects of hygroscopic expansion and shrinkage, the dead layer of bark was removed. SRV data were recorded at 10-min intervals using a data logger (DL 15, HOBO, USA) throughout 2020. The raw dendrometer data were time-aligned and cleaned using ‘*datacleanr*’ R package (Hurley et al. 2022), with erroneous shifts and jumps corrected using the ‘*treenetproc*’ R package (Knüsel et al. 2021).

The ‘zero-growth’ concept was applied to separate continuous stem radius measurements into growth-induced irreversible stem expansion (GRO) and tree water deficit (TWD)-induced reversible stem shrinkage (TWD). This approach assumes no growth during the periods of stem shrinkage (Zweifel et al. 2016). Accumulative growth was recorded when the stem radius exceeded its previous maximum (GRO > 0), while stem shrinkage or expansion below the maximum was defined as periods of TWD. Thus, dendrometer measurements reflect the physiological response of trees to climate conditions in terms of both growth and water status (Zweifel et al. 2005, 2016). The GRO and TWD values, recorded at 10-min intervals, were aggregated to a daily scale for each tree and year. We defined the probability of GRO occurrence as growth (1, GRO rate > 0 μm day^−1^) and no growth (0, GRO rate = 0 μm day^−1^). Considering the highly variable absolute annual growth between individual trees, we calculated relative daily growth rates (%) by normalizing the absolute daily GRO rate (μm day^−1^) to the total annual growth for each tree and year (Zweifel et al. 2021).

### Microcore collection and xylem formation

To investigate the intra-annual dynamics of vessel and fiber formation, wood microcores were collected from five trees of each species in 2020 using a Trephor microborer (Rossi et al. 2006*a*). After removing the outer bark, microcores of 2 mm in diameter and 1.5 cm in length were extracted at a stem height of about 1.3 m. Microcores with intact cambium were immersed in 70% ethanol and stored at 5 °C (Rossi et al. 2006*b*). The samples were then dehydrated with grading ethanol and D-limonene and embedded in paraffin blocks. Cross-sections of 15–20 μm thickness were cut with a rotary microtome (DM2245 Leica) and fixed on microscope slides. The slides were stained with a solution of 1% Safranin for 2 h, followed by 5% Astra blue for 1 min. Cross-sections were imaged at 200× magnification using a digital camera mounted on a light microscope (DM 2500, Leica). Polarized light was applied to distinguish xylem differentiation phases.

**Figure 1:**
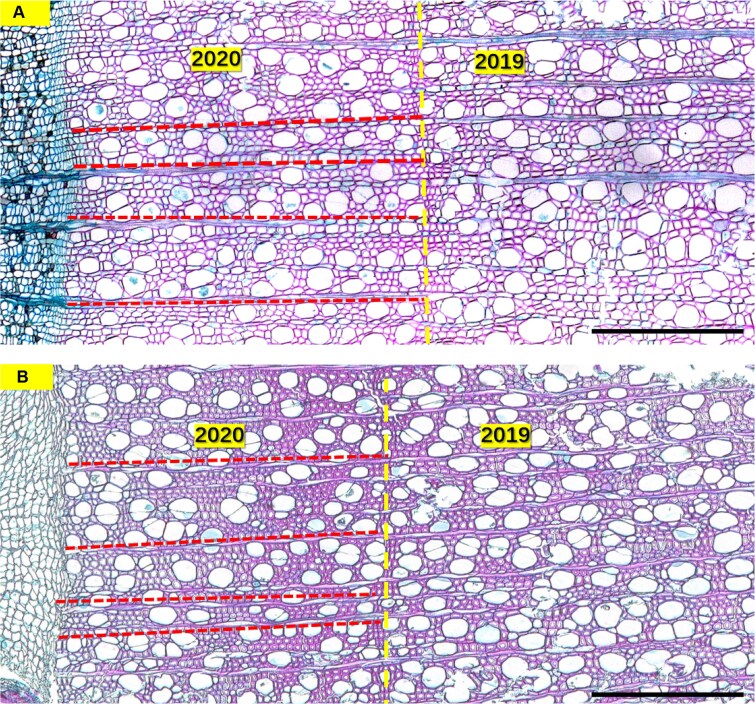
Cross-sections of *S. pteropetiolata* (A) and *S. noronhae* (B). Vertical lines denote the ring boundaries. Scale bar = 500 μm. The area between horizontal lines denotes a radial area delimited by the ray parenchyma on its tangential sides.

Cell anatomical measurements of mature vessels and fiber cells were conducted using high-resolution images with the ImageJ software (http://rbs.info.nih.gov/ij/). We measured the length of xylem cell differentiation zones on each cross-section during 2020 for both species. The number of vessels and fibers at different developmental stages was counted along three radial profiles per sample. Each radial area was delimited by the ray parenchyma on its tangential sides. The number of vessels or fiber cells in each radial file was calculated by dividing the total cell numbers by the corresponding width of the radial area (Figure 1). Finally, biweekly counts of the two types of cells (fiber cells and vessels) in each differentiation zone (EZ, enlarging zone; WZ, wall thickening zone; MZ, mature zone) were recorded and standardized using the ‘*CAVIAR*’ R package (Rathgeber et al. 2011). The cumulative sums of enlarging cells (EWMZ = EZ + WZ + MZ), wall thickening cells (WMZ = WZ + MZ) and mature cells (MZ) were quantified as the relative growth to the total annual growth and subsequently fitted using shape-constrained additive models (SCAMs). Additionally, the relative entry rates of cells into the differentiation zone were calculated from cumulative sums of differentiating and mature cells and then fitted by generalized additive models (Cuny et al. 2013, Pya and Wood 2015, Pérez-de-Lis et al. 2022) using the ‘*mgcv*’ R package (Wood 2017).

### Statistical methods

We used linear models to analyze relative daily growth rates (%, normalized by annual growth) in relation to environmental variables, including daily minimum temperature (DMT), VPD, SWC and their two-way interactions. Given the high correlation among daily minimum, maximum and mean temperatures, only DMT was considered for analysis, as most species mainly grow at night when DMT typically occurs (Zweifel et al. 2021). Besides, we applied the square root transformation to the relative daily growth rate because of its non-normality. We also interpolated relative daily growth rates in relation to the essential environmental conditions and fitted them using the local polynomial regression (loess) function. Contour diagrams were generated with the ‘*gridextra*’ and ‘*reshape2*’ R packages (Wickham 2016, Auguie and Antonov 2017). Uncertainty analysis was conducted through a bootstrapping procedure, randomly resampling 1000 times and calculating the coefficient of variation (Zweifel et al. 2021).

To evaluate the impact of environmental variables on the growth of fibers and vessels during different stages of xylogenesis, we fitted generalized linear models (GLMs), with the relative rates of cell differentiation being response variables, and SWC, VPD, DMT, species, phases of cell differentiation, and their two-way interactions as explanation variables. We selected the beta family for the models using the ‘*betareg*’ R package (Harrison 2015, Zeileis et al. 2016). From the results of GLMs, we calculated derivatives of the predicted values to assess the relative rates of cell formation in response to environmental variables (DMT, SWC, VPD). We tested for collinearity in all models using the variance inflation factor (VIF). In the case of collinearity (VIF > 10), we retained the variable strongly correlation with the response variable. Additionally, we checked model assumptions (normality of residuals, homogeneity of variance, multicollinearity) using the *‘check_model*’ function in the ‘*performance*’ R package (Lüdecke et al. 2021). All analyses were conducted in R software (version 4.3.1, R Core Team, 2023).

## Results

### Spatial distribution of study species

**Figure 2:**
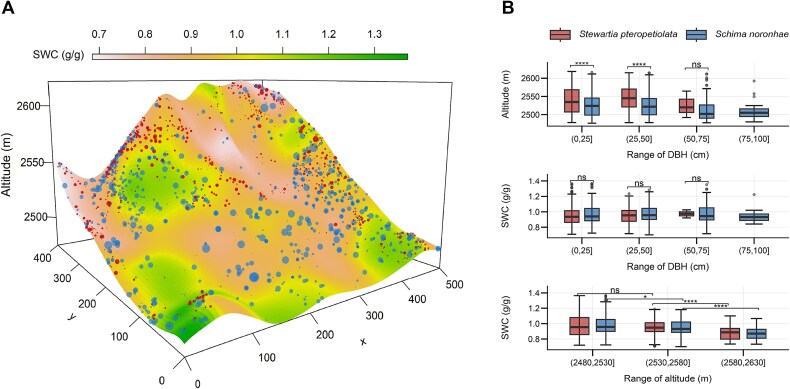
Spatial distribution of *S. pteropetiolata* and *S. noronhae* trees in the 20-ha permanent plot. Colors correspond to volumetric soil water content (SWC, g/g), which was interpolated from 20 × 20 m plots (A). Box plots (B) display the ranges of altitude and SWC across tree size (diameter at breast height) classes, and the ranges of SWC across altitudes. Significance among different tree size classes and altitudes were determined by Wilcoxon test. *n.s.*, non-significant; ^*^P < 0.05; ^**^P < 0.01; ^***^P < 0.001; ^****^P < 0.0001.

The two study species showed distinct spatial distribution along the elevation and moisture gradient, according to the survey data from a 20-ha permanent plot located less than 1 km from our study site. *Schima noronhae* is predominantly found at lower altitudes, while *S. pteropetiolata* is more common at higher elevations. Although there is no significant difference in SWC across the habitat ranges of these two species within the 20-ha plot, SWC decreases with increasing altitude in the areas where both species coexist (Figure 2).

### Environmental drivers of stem growth

**Figure 3:**
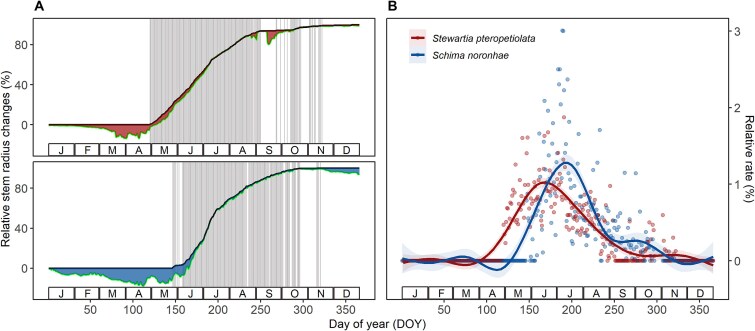
Seasonal variations of relative stem radius changes (A, lower and fluctuating lines) and relative daily growth rate (B) for *S. pteropetiolata* and *S. noronhae* in 2020. Cumulative growth (A, upper and smooth lines), stem shrinkage induced by tree water deficit (TWD) (A, shaded areas), averaged from three individuals per species, were developed from the zero-growth concept (ZG model). Vertical bins in figure a represented the days of growth-induced irreversible expansion (GRO > 0). Relative daily growth rates (B, solid dots) were fitted with generalized additive models (GAMs).

The intra-annual stem growth showed a distinct seasonality with species-specific patterns (Figure 3). Prior to the onset of growth, both species experienced extended periods of stem contraction due to low soil water availability. However, *S. noronhae* experienced a more prolonged and severe water deficit than *S. pteropetiolata* during the early growing season. *Stewartia pteropetiolata* began growth in late April, while *S. noronhae* started to grow in late May. Both species ceased growth in mid-November (Figure 3A). Notably, *S. noronhae* exhibited rapid growth, with peak GRO rate occurring 1 month later (July) than *S. pteropetiolata* (Figure 3B).

**Figure 4:**
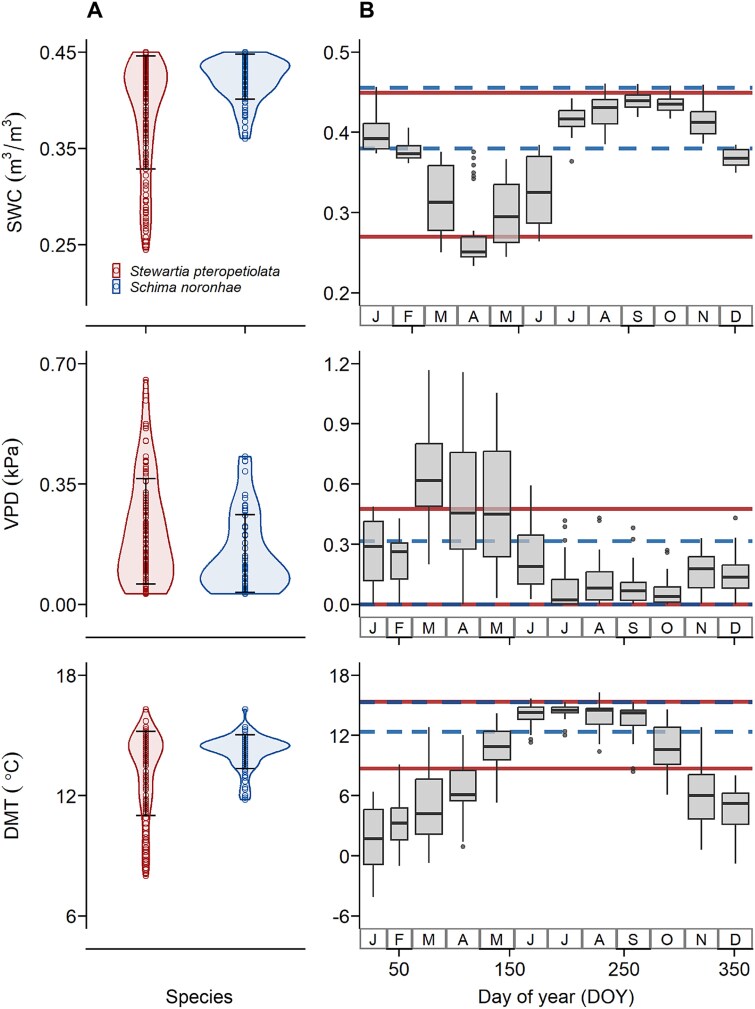
Comparison of environmental variables (SWC, soil water content; VPD, vapor pressure deficit; DMT, daily minimum temperature) during the days with irreversible growth (GRO > 0) for *S. pteropetiolata* and *S. noronhae*. Box plots (B) display monthly variations of environmental variables in 2020, the horizontal lines correspond to the range within which 90% of the data from violin plots (A) were concentrated.

Climatic conditions in 2020 exhibited pronounced seasonal variations (Figure 4B). Analysis of climate variables on days with growth (GRO rate > 0 μm) indicated that stem growth mainly occurred during the rainy season (from May to October), which was characterized by high temperature (DMT ranging from 9 to 15 °C) and SWC (ranging from 0.27 to 0.45 m^3^ m^−3^), coupled with low VPD (ranged from 0 to 0.45 kPa). Notably, the growth events of *S. pteropetiolata* occurred within a broader range of climatic conditions, with lower peak values compared with *S. noronhae* (Figure 4A).

**Figure 5:**
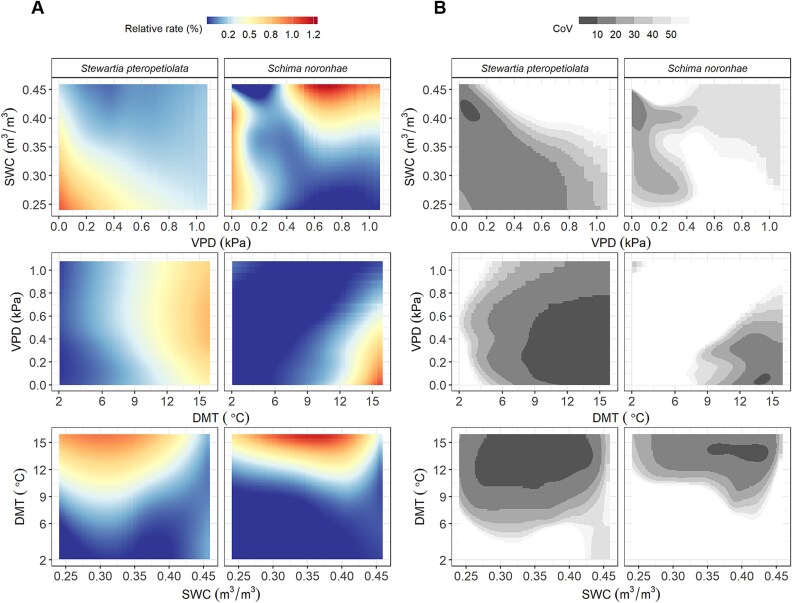
Relative daily growth rate in relation to soil water content (SWC), vapor pressure deficit (VPD) and daily minimum temperature (DMT). Relative daily growth rate (A) is color-coded and ranges from no growth (0%) via little growth (0.5%) to high growth (1.2%). The coefficient of variation (CoV) of the uncertainty analysis (B) indicated the robustness of the results between very good (values < 10), good (10 to < 20) and satisfactory (20–50), to poor (> 50).

The results of linear models indicated that the daily growth rates for both species were influenced by VPD, SWC and DMT (Table S1 and Figure S1 available as Supplementary data at *Tree Physiology* Online). Both species showed high relative growth rates across wide ranges of SWC when VPD was low (< 0.2 kPa) and DMT was high (> 12 °C). However, *S. pteropetiolata* maintained substantial growth even under conditions of low SWC, high VPD and low DMT. *Schima noronhae* can sustain relatively high rates under high VPD, but only when SWC was higher than 0.4 m^3^ m^−3^ (Figure 5A). The uncertainty for growth within the specific climatic conditions was low (Figure 5B).

### Environmental drivers of cell formation

**Figure 6:**
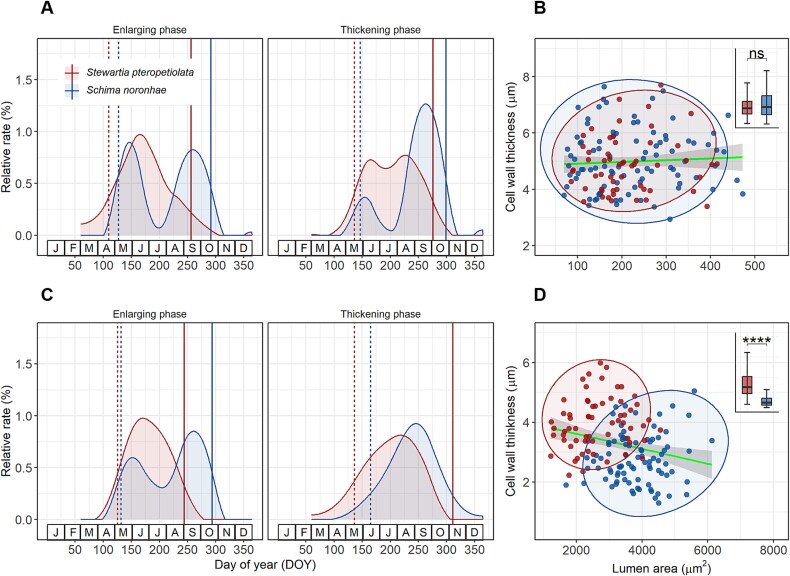
Relative rates of cell differentiation and xylem anatomical features (cell lumen area and cell wall thickness) for fiber cells (A, B) and vessels (C, D). The onset (A, C; dotted line) and cessation (A, C; solid line) for each phase are displayed as well. The linear relationship between cell lumen area and cell wall thickness are indicated by the line with 95% confidence interval (shaded) fitted from the sample points (B, D). Differences in the ratio of lumen area to cell wall thickness between the two studied species were tested by Student’s *t*-test and displayed by box plots (B, D). *ns*, non-significant; ^****^P < 0.0001.

The SCAMs revealed that the kinetics of xylem formation were species-specific (Figure S2 available as Supplementary data at *Tree Physiology* Online). Although there were no significant anatomical differences between the fiber cells of two species, *S. noronhae* had larger vessels with thinner cell walls (Figure 6B and D) and its vessels experienced longer enlarging phase and shorter wall thickening phase than *S. pteropetiolata* (Figure 6C). From June to July, the relative rate of cell enlarging decreased for both fiber cells and vessels, while the cell-wall thickening rate decreased for fiber cells but increased for vessels (Figure 6A and C). Similarly, there was no obvious relationship between cell wall thickness and lumen area for fiber cells, but a slight negative correlation was found for vessels (Figure 6B and D).

**Figure 7:**
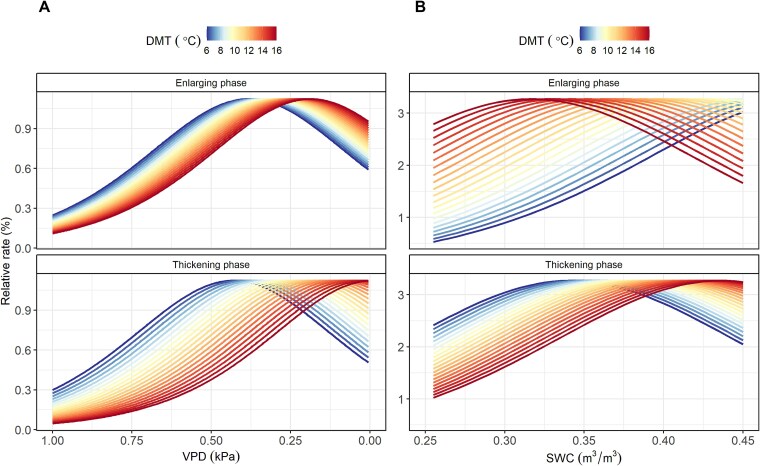
Relative rates of cell differentiation (cell enlarging and cell wall thickening) in relation to soil water content (SWC), vapor pressure deficit (VPD) and daily minimum temperature (DMT) for fiber cells (A, left panels) and vessels (B, right panels).

The results of GLMs indicated that DMT was the primary driver of xylem formation kinetics (Table S2 available as Supplementary data at *Tree Physiology* Online). The differentiation of fiber cells was mainly influenced by DMT and VPD, while the differentiation of vessels was primarily driven by changes in SWC and DMT (Table S2 available as Supplementary data at *Tree Physiology* Online). For fiber cells, the relative rate of cell enlarging and cell wall thickening increased with rising DMT when VPD roughly ranged from 0 to 0.25 kPa, indicating similar responses of these two development phases to DMT (Figure 7A). Conversely, the responses of cell enlarging and cell wall thickening to DMT were opposite for vessels. For example, when SWC ranged from 0.25 to 0.38 m^3^ m^−3^, the relative rate of cell enlarging increased with higher DMT, while the relative rate of cell wall thickening decreased (Figure 7B).

## Discussion

### Response of intra-annual stem growth to environment

In this study, we quantified the intra-annual dynamics of radial growth and xylem formation in two dominant canopy species of a subtropical evergreen broad-leaved forest. Our findings demonstrated that DMT and SWC exert greater influences on tree growth than VPD in our study region. The importance of DMT can be attributed to the evidence that most tree species mainly grow at night (Zweifel et al. 2021), when DMT typically occurs. Our results revealed that radial growth rates were positively correlated with DMT (Table S1 and Figure S1C available as Supplementary Data at  *Tree Physiology* Online). When sufficient moisture is available, warmer minimum temperatures enhance metabolic efficiency (Parent and Tardieu 2012) at the beginning of the growing season, stimulate cambial activity (Cabon et al. 2020*b*), initiate stem growth (Huang et al. 2020) and increase growth rates (Zhou et al. 2023).

Other studies have highlighted the key roles of VPD and soil water potential in regulating tree water status and growth (Steppe et al. 2006, Novick et al. 2016, Carminati and Javaux 2020). The role of soil moisture in altering some physiological functions in trees has been particularly emphasized (Muller et al. 2011, Klein et al. 2014). Our results showed that as elevation increases, both species tend to occur at lower SWC (Figure 2). Besides, the radial growth rates were negatively correlated with SWC (Table S1 and Figure S1A available as Supplementary Data at  *Tree Physiology* Online). This result aligns with previous findings that the growth of *S. noronhae* was hindered by excessive water supply during the growing season (Xu et al. 2024). Trees in moist forests grew better during the dry season than in the wet season (Huang et al. 2018), while excessive rainfall during the growing season inhibited the radial growth of moist forest trees (Fontana et al. 2019). Moreover, tree growth has been observed in moderately dry soils under conditions of low VPD (Zweifel et al. 2021). In view of the above, we conclude that low temperature, rather than moisture, may be the limiting growth factor in our studied moist forests.

Our results further revealed that the response of intra-annual growth to environmental conditions aligned with the spatial ecological niches of the studied species in terms of temperature, but not regarding moisture conditions. Despite similar SWC across the habitats of both species, *S. noronhae*, which occupies lower elevations, exhibited high relative growth rates within a narrower range of climatic conditions characterized by higher DMT. Conversely, *S. pteropetiolata*, found at higher elevations, demonstrated growth across a broader range of climatic conditions with lower DMT. (Figures 2, 4A and 5A). These results suggest that *S. noronhae* is more sensitive to environmental conditions and has a higher heat requirement than *S. pteropetiolata*, which likely explains the 1-month delay in the onset of growth for *S. noronhae* (Figure 3). Consequently, the primary growth period of *S. noronhae* (from June to September) was characterized by higher DMT (the median close to 14 °C) and rapidly increasing SWC (Figure 4B).

### Cell formation and environmental responses

Temperature and soil moisture conditions were the main drivers of the kinetics of xylem cell formation, especially for their vessels’ characteristics. The relationship between DMT and the development of different cell types (fiber cells and vessels) differed between species. Besides, the development of vessels mainly depended on SWC and DMT (Table S2 available as Supplementary data at *Tree Physiology* Online). At lower elevations, *S. noronhae* produced wider vessels with thinner walls within a narrower range of favorable temperatures and soil moisture during the rainy season, while at higher elevations, *S. pteropetiolata* developed smaller vessels with thicker cell walls (Figures 2, 4A and 6D). Previous studies have shown that broadleaved tree species can alter the hydraulic architecture, particularly the size and frequency of vessels, to ensure hydraulic safety and survival within their natural distribution range (Cosme et al. 2017, Hacke et al. 2017, Islam et al. 2019, Silva et al. 2020, Nascimento et al. 2024).

Cell size across the tree ring has been considered closely tied to the temporal dynamics of xylogenesis, with xylem cell diameter largely determined by the duration of the enlargement phase (Buttò et al. 2019, Li et al. 2021). Based on the environmental conditions required for stem growth in *S. noronhae*, the relative rate of cell enlargement decreased, and the relative rate of cell wall thickening increased during vessel development (Figure 7B). This could result in a longer cell enlargement phase and a shorter cell wall-thickening phase (Figure 6C). As a result, *S. noronhae* develops vessels with larger lumens and thinner cell walls (Figure 6D).

Larger vessels are usually associated with higher fractions of parenchyma (Morris et al. 2018) and greater water transport efficiency (Tyree and Zimmermann 2013), which might be beneficial for *S. noronhae* to achieve higher growth rates despite the constraints imposed by low temperatures and a short growing season. Furthermore, *S. noronhae* began growing about 1 month later than *S. pteropetiolata*, an adaptation likely aimed at reducing the risk of xylem embolism caused by late-spring freezing in large vessels (Wang et al. 1992, Pérez-de-Lis et al. 2016). In contrast, *S. pteropetiolata*, with thicker vessel walls and smaller vessels, likely has thicker and less porous pit membranes and higher embolism resistance (Jacobsen et al. 2005, Jansen et al. 2009, Plavcová et al. 2011, Torres-Ruiz et al. 2017) to minimize the damage because of frost-induced cavitation and to maintain the hydraulic function of xylem (Fisher et al. 2007, Schreiber et al. 2015).

## Conclusions

This study investigated the inter-annual stem growth and cell formation of two subtropical tree species in Southwest China, as well as their responses to seasonal climate variability. We observed distinct seasonal patterns of stem growth and cell formation in both species, likely linked to their wood anatomical features and stress tolerance. *Stewartia pteropetiolata*, found at higher elevations, sustained growth under colder and drier conditions, developing smaller vessels with thicker cell walls. In contrast, *S. noronhae*, found at lower elevations, produced wider vessels with thinner walls while maintaining relatively higher growth rates within a narrower range of favorable temperatures and soil moisture during the rainy season. Our findings underscore that species with specific wood anatomical features occupy specific ecological niches and exhibit unique intra-annual growth patterns, likely reflecting their tolerances to drought and cold conditions. These findings help to clarify how wood anatomical traits shape the distributions of evergreen broad-leaved tree species in subtropical moist forests and their responses to climate variability. Additionally, the inter-specific seasonal variability in stem growth and xylogenesis should be taken into account when modeling forest dynamics and predicting forests’ response to climate change.

## Supplementary Material

figureS1_tpaf020

figureS2_tpaf020

Supplementary_R2_tpaf020

## Data Availability

All experimental data are available upon request to the corresponding author.
