# Supplementary figures and images for: Intra-annual stem radius growth and cell formation of two diffuse-porous tree species in a subtropical forest in Southwest China
Source: Tree Physiol. 2025 Feb 17;45(3):tpaf020. doi: 10.1093/treephys/tpaf020 (PMC11937825; doi:10.1093/treephys/tpaf020)

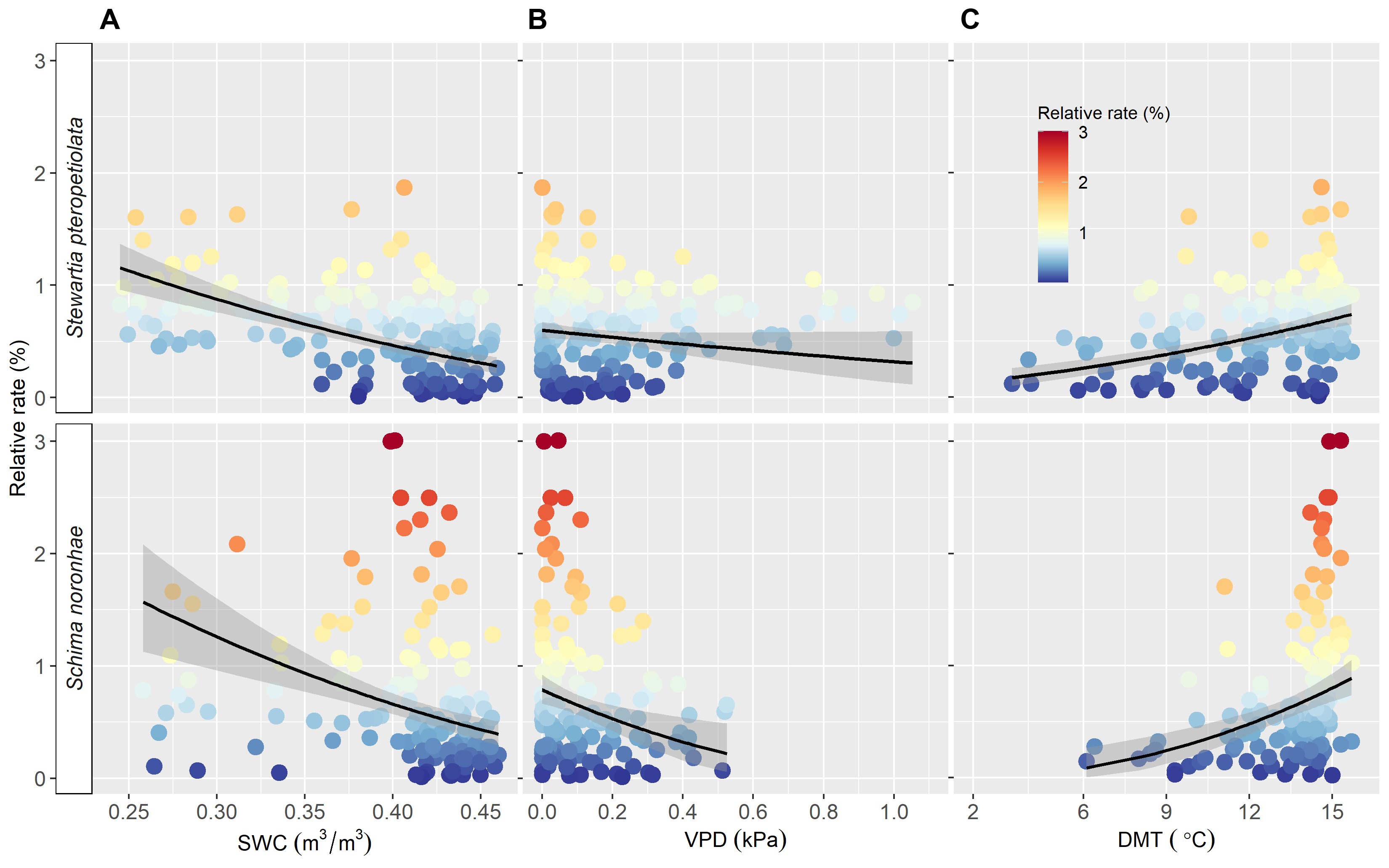

Supplement: figureS1_tpaf020 [file figures1_tpaf020.jpeg]

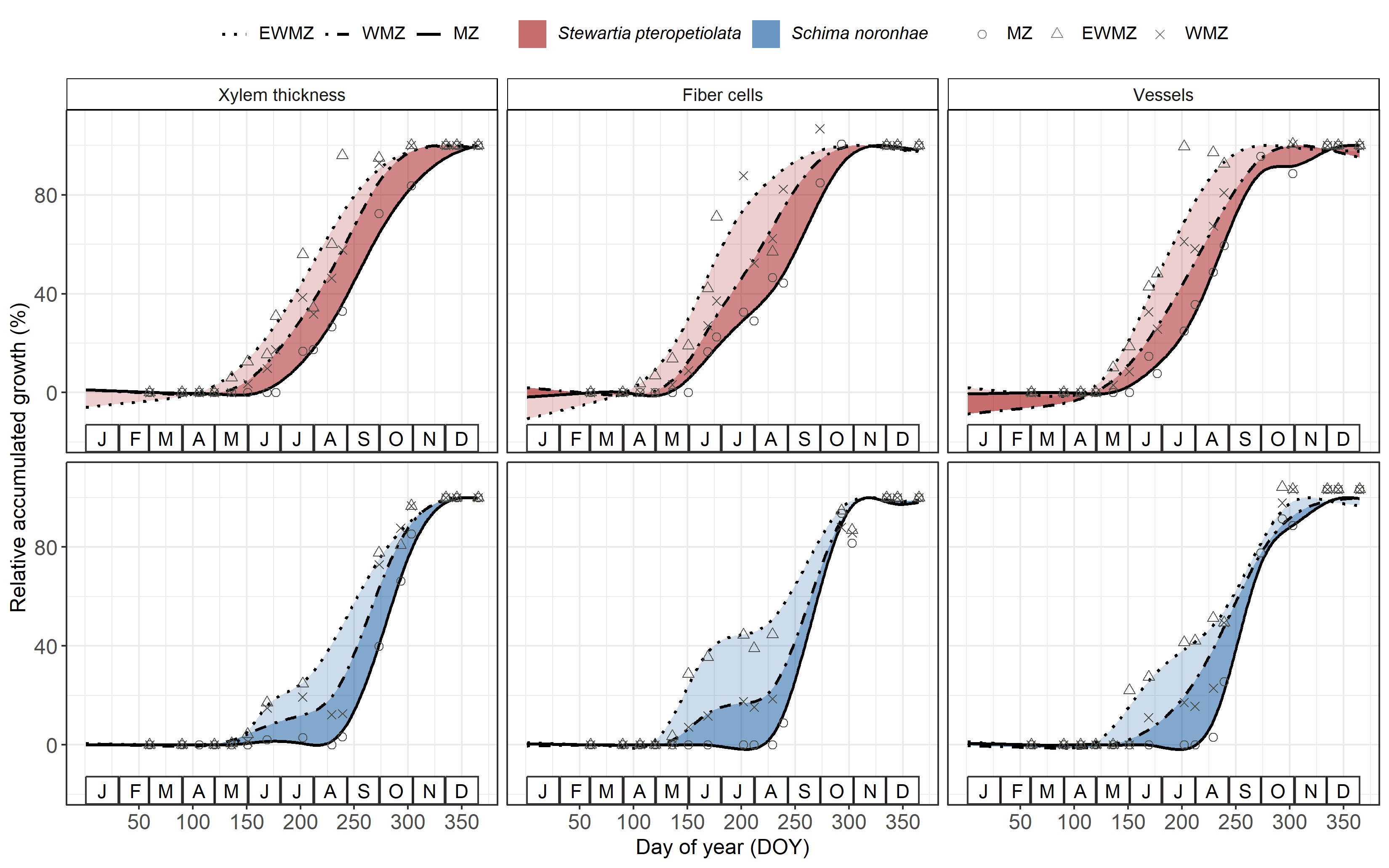

Supplement: figureS2_tpaf020 [file figures2_tpaf020.jpeg]
