# Supplementary material for: Intra-annual stem radius growth and cell formation of two diffuse-porous tree species in a subtropical forest in Southwest China
Source: Tree Physiol. 2025 Feb 17;45(3):tpaf020. doi: 10.1093/treephys/tpaf020 (PMC11937825; doi:10.1093/treephys/tpaf020)
Supplement: Supplementary_R2_tpaf020 [file supplementary_r2_tpaf020.docx]

**Supplemental Materials**

**Table S1.** Analysis of variance results for the relative daily growth rate of *S. pteropetiolata* and *S. noronhae*. Soil water content (SWC), vapor pressure deficit (VPD), daily minimum temperature (DMT), and their two-way interactions were considered as explanation variables in the linear models. Df = degrees of freedom, N = number of observations, R^2^ = explanatory power of the model, R^2^ Adj = adjusted R^2^, AIC = Akaike Information Criterion, BIC = Bayesian Information Criterion, RMSE = Root mean square errors.

|  | | *S. pteropetiolata* | | | | *S. noronhae* | | | | | | |
| --- | --- | --- | --- | --- | --- | --- | --- | --- | --- | --- | --- | --- |
|  | | | Df | F value | Pr(>F) | |  | | Df | F value | Pr(>F) | |
| SWC | | | 1 | 40.98 | < 0.001 | |  | | 1 | 6.13 | < 0.05 | |
| VPD | | | 1 | 5.27 | < 0.05 | |  | | 1 | 22.95 | < 0.001 | |
| DMT | | | 1 | 67.27 | <0.001 | |  | | 1 | 29.83 | < 0.001 | |
| SWC × VPD | | | 1 | 0.77 | 0.38 | |  | | 1 | 6.86 | < 0.01 | |
| VPD × DMT | | | 1 | 6.52 | < 0.05 | |  | | 1 | 2.41 | 0.12 | |
| SWC × DMT | | | 1 | 0.88 | 0.35 | |  | | 1 | 3.50 | 0.06 | |
| N | 163 | | | | | | | 137 | | | |  |
| R^2^ | 0.47 | | | | | | | 0.33 | | | |  |
| R^2^ Adj. | 0.45 | | | | | | | 0.30 | | | |  |
| AIC | 39.2 | | | | | | | -476.9 | | | |  |
| BIC | 64.0 | | | | | | | -453.5 | | | |  |
| RMSE | 0.19 | | | | | | | 0.29 | | | |  |

**Table S2.** Analysis of deviance results for the growth contribution of fiber cells and vessels. Soil water content (SWC), vapor pressure deficit (VPD), daily minimum temperature (DMT), species, phases of cell differentiation, the interaction between DMT and species, and the interaction between DMT and phases were considered as explanation variables in the generalized linear models. Df = degrees of freedom, N = number of observations, R^2^ = explanatory power of the model, AIC = Akaike Information Criterion, BIC = Bayesian Information Criterion, RMSE = Root mean square errors.

|  | | Fiber cells | | | | | | | Vessels | | | | | | | |
| --- | --- | --- | --- | --- | --- | --- | --- | --- | --- | --- | --- | --- | --- | --- | --- | --- |
|  | | | Df | | Chisq | | Pr(>Chisq) | | |  | | Df | Chisq | Pr(>Chisq) | | |
| DMT | | | 1 | | 13.24 | | < 0.001 | | |  | | 1 | 19.12 | < 0.001 | | |
| phase | | | 2 | | 16.57 | | < 0.001 | | |  | | 2 | 17.45 | < 0.001 | | |
| specie | | | 1 | | 10.49 | | < 0.01 | | |  | | 1 | 7.46 | < 0.01 | | |
| VPD | | | 1 | | 11.46 | | <0.001 | | |  | | 1 | 0.01 | 0.95 | | |
| SWC | | | 1 | | 2.19 | | 0.14 | | |  | | 1 | 13.14 | < 0.001 | | |
| DMT × phase | | | 2 | | 6.01 | | < 0.05 | | |  | | 2 | 8.58 | < 0.05 | | |
| DMT ×specie | | | 1 | | 6.48 | | < 0.05 | | |  | | 1 | 8.31 | < 0.01 | | |
| N | 57 | | | | | | | | | | 53 | | | | |  |
| R^2^ | 0.66 | | | | | | | | | | 0.60 | | | | |  |
| AIC | -47.6 | | | | | | | | | | -30.6 | | | | |  |
| BIC | -29.2 | | | | | | | | | | -12.8 | | | | |  |
| RMSE | 0.17 | | | | | | | | | | 0.17 | | | | |  |
|  | | | |  | |  | |  | | | | | | |  |  |

**Figure S1.** Relationships between relative daily growth rates and each of the variables soil water content (A), vapor pressure deficit (B), and daily minimum temperature (C). Black lines indicate the fitted values, with lower and upper confidence intervals at 0.95 displayed. Relative daily growth rates (solid circles) are color-coded from dark blue (low growth rates) to red (high growth rates).


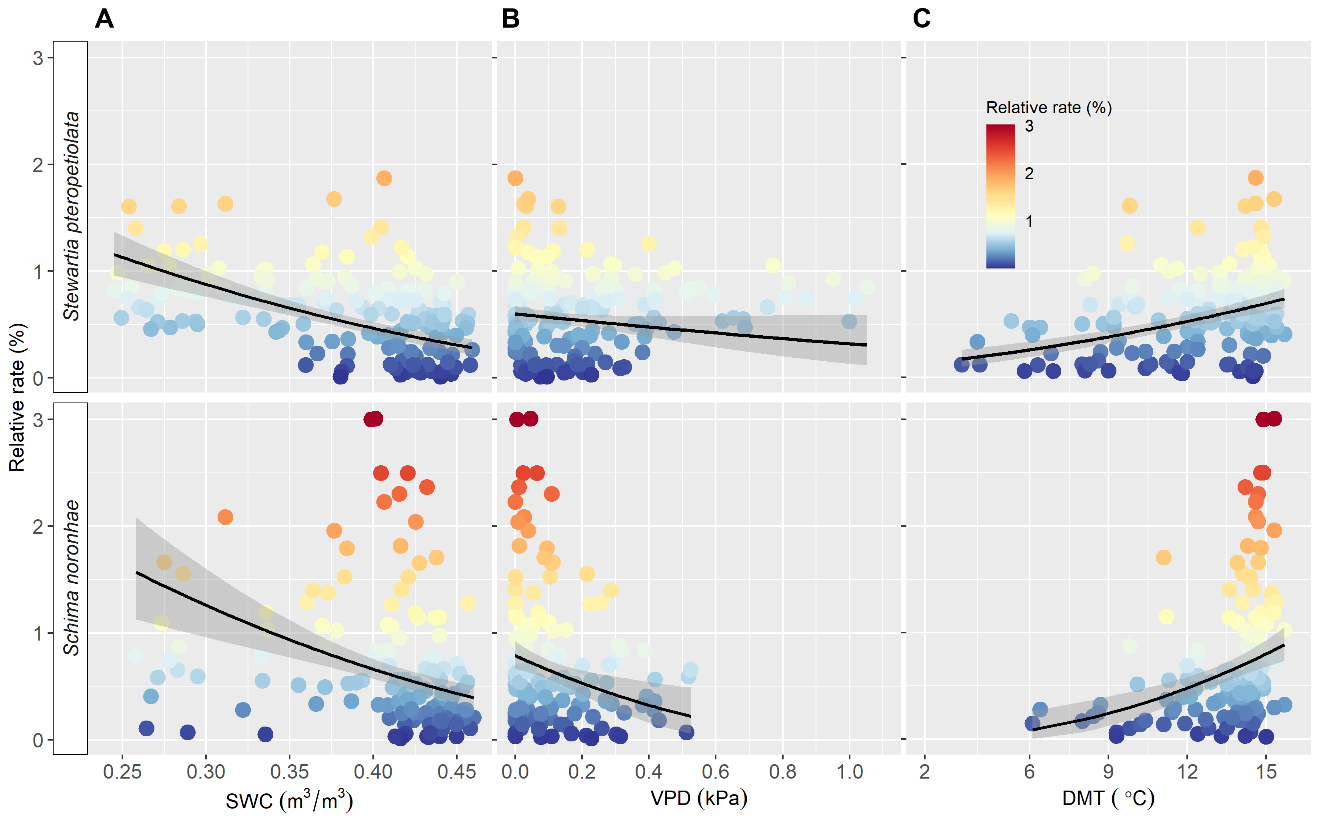


**Figure S2.** The kinetics of xylem thickness formation, fiber cell formation, and vessel formation. Observations were averaged from five trees and normalized by annual xylem thickness or the total number of fiber cells or vessels (in percentage; the annual xylem thickness or the total number of cells equal to 100%). Line type and point shape correspond to the fitted values and observations at different formation stages, respectively. Dotted lines or triangles indicate the cumulative sums of enlarging cells (EWMZ); dot-dash lines or crosses indicate the cumulative sums of wall thickening cells (WMZ); solid lines or circles indicate the cumulative sums of mature cells (MZ). Color corresponds to species (red = *Stewartia pteropetiolata*, blue = *Schima noronhae*).


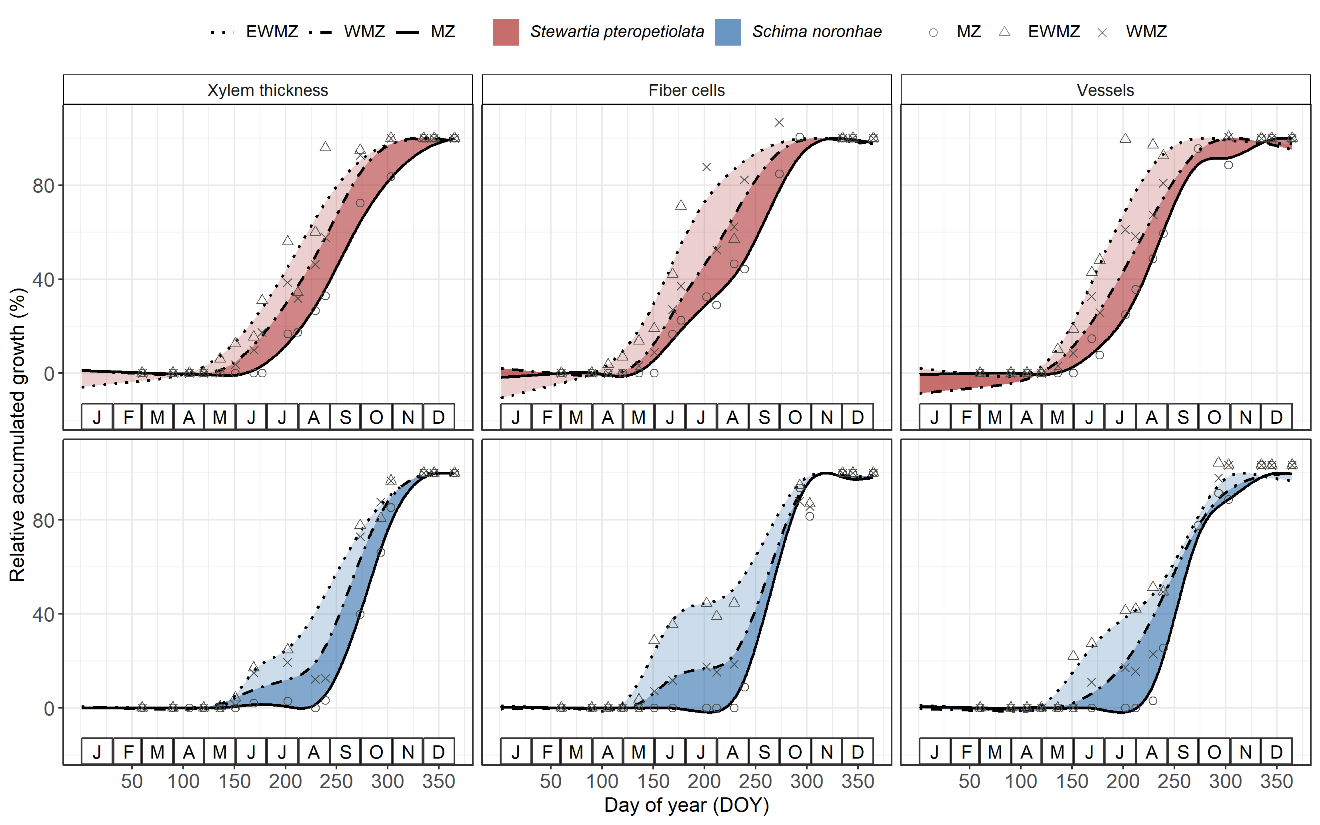


.
